# Gadd45β promotes regeneration after injury through TGFβ-dependent restitution in experimental colitis

**DOI:** 10.1038/s12276-019-0335-y

**Published:** 2019-10-30

**Authors:** Jung Hwan Hwang, Tae-Hwan Kim, Yong-Hoon Kim, Jung-Ran Noh, Dong-Hee Choi, Kyoung-Shim Kim, Eun-Young Lee, Byoung-Chan Kim, Myung Hee Kim, Ho Kim, Tae Geol Lee, Jong-Soo Lee, Chul-Ho Lee

**Affiliations:** 1 Laboratory Animal Resource Center, Korea Research Institute of Bioscience and Biotechnology (KRIBB), Yueong-gu, Daejeon, 34141 Korea; 2 University of Science and Technology, 125 Gwahak-ro, Yueong-gu, Daejeon, 34141 Korea; 3 College of Veterinary Medicine, Chungnam National University, Daejeon, Korea; 4 Infection and Immunity Research Laboratory, Metabolic Regulation Research Center, KRIBB, Daejeon, Korea; 5 Department of Life Science, College of Natural Science, Daejin University, Pocheon-si, Gyeonggi-do, Korea; 6 Center for Nano-Bio Measurement, Korea Research Institute of Standard and Science, 267 Gajeong-ro, Yuseong-gu, Daejeon, 34113 Korea

**Keywords:** Acute inflammation, Mechanisms of disease

## Abstract

Dysregulated immune responses and impaired function in intestinal epithelial cells contribute to the pathogenesis of inflammatory bowel disease (IBD). Growth arrest and DNA damage-inducible 45 beta (Gadd45β) has been implicated in the pathogenesis of various inflammatory symptoms. However, the role of Gadd45β in IBD is completely unknown. This study aimed to evaluate the role of Gadd45β in IBD. Gadd45β-KO mice exhibited drastically greater susceptibility to dextran sulfate sodium (DSS)-induced colitis and mortality than C57BL/6J mice. Bone marrow transplantation experiments revealed that Gadd45β functions predominantly in the intestinal epithelium and is critical during the recovery phase. Gadd45β regulates the TGF-β signaling pathway in colon tissue and epithelial cells by inhibiting Smurf-mediated degradation of TGF-β receptor type 1 via competitive binding to the N-terminal domain of Smad7. Furthermore, these results indicate that the Gadd45β-regulated TGF-β signaling pathway is involved in wound healing by enhancing epithelial restitution. These results expand the current understanding of the function of Gadd45β and its therapeutic potential in ulcerative colitis.

## Inflammatory bowel disease: Bolstering the intestinal barrier

A signaling molecule that prevents inflammatory damage in an animal model of ulcerative colitis offers a promising therapeutic target. The molecular drivers of this form of inflammatory bowel disease remain poorly understood, but the associated damage to the intestinal epithelium is primarily due to uncontrolled immune cell activity. Jung Hwan Hwang of the Korea Research Institute of Bioscience and Biotechnology, Daejeon, and coworkers have now demonstrated that a protein called Gadd45β helps to reduce inflammatory damage to the epithelial barrier. They showed that a mouse model of chemically induced ulcerative colitis exhibited more severe disease symptoms and higher mortality when these animals also lacked Gadd45β. This protein is generally known to modulate immune cell activity response, but in this disease model, the authors primarily observed activity within intestinal epithelial cells, where it appears to facilitate wound healing.

## Introduction

Inflammatory bowel disease (IBD) manifests as chronic intestinal inflammation and structural and functional disruptions of the gastrointestinal tract^1^. IBD exists in two clinical forms: Crohn’s disease (CD) and ulcerative colitis (UC). In CD, inflammation can occur in any part of the gastrointestinal tract. Ulcerative colitis is, however, restricted to the large intestine^2^. The incidence and prevalence of IBD have recently increased steadily in developing and developed countries;^3^ however, its pathogenesis has remained elusive. The etiology of IBD involves multiple factors, including host genetic susceptibility, the immune response, environmental triggers, and luminal microbes^4,5^. Susceptibility genes have been identified through genome-wide association studies;^6,7^ however, new genetic factors warrant elucidation.

Gadd45β was initially considered to be a myeloid differentiation primary response gene (*MyD118*) of the Gadd45 family, which includes *Gadd45α*, *Gadd45β*, and *Gadd45γ*^8,9^. These genes are expressed in response to various physiological and environmental stressors and regulate DNA repair, cell cycle arrest, cell survival, and apoptosis, depending on cell type^9^. *Gadd45β* is expressed in response to stressors including various stress factors, such as interleukin-6 (IL-6), tumor necrosis factor-α (TNF-α), transforming growth factor-beta (TGF-β), lipopolysaccharide (LPS), and drugs^10–14^. However, the functions of Gadd45β depend on the cell type and environment. Indeed, Gadd45β promotes TGF-β-mediated cell death in some cells but inhibits TNF-α-induced apoptosis in TNF-α-treated T cell hybridomas by inhibiting the JNK response to TNFα via a direct interaction with the upstream kinase MKK7. Gadd45β is involved in innate and adaptive immunity. In an experimental sepsis model, Gadd45β-KO mice exhibited reduced myeloid cell recruitment to the peritoneal cavity upon LPS stimulation^15^. Moreover, the macrophages and granulocytes of Gadd45α/β double-KO mice exhibited reduced migratory efficiency in chemotactic assays^15^. Gadd45β promotes Th1 responses by inducing IFN-γ secretion upon T-cell receptor stimulation or in response to IL-12 and IL-18, which are involved in Th1 differentiation^16^. Despite evidence for the immunoregulatory role of Gadd45β, its roles in IBD are unknown.

In this study, we investigated the role of Gadd45β in intestinal homeostasis using rodents lacking Gadd45β and control wild-type (WT) C57BL/6J mice to establish a dextran sulfate sodium (DSS)-induced colitis model mimicking the clinical pathogenesis of UC.

## Materials and methods

### Antibodies and reagents

Antibodies (Abs) against phospho-Jnk1/2, total-Jnk1/2, phospho-PKB (pS473), total-PKB, phospho-p38, total-p38, phospho-Smad2, total-Smad2, phospho-Smad3, total-Smad3, PCNA, and α-tubulin were purchased from Cell Signaling (Beverly, MA, USA). An antibody against Gadd45β was obtained from Aviva Systems Biology (San Diego, CA, USA). Antibodies against β-actin, HA, Myc, and GST were purchased from Santa Cruz Biotechnology (Dallas, TX, USA). Antibodies against V5 and Flag were purchased from Invitrogen (Carlsbad, CA, USA). Cy3-conjugated donkey anti-mouse IgG and Alexa 488-conjugated goat anti-rabbit IgG antibodies were obtained from The Jackson Laboratory (Bar Harbor, ME, USA) and Invitrogen (Waltham, MA, USA), respectively. An anti-Strep MAB-classic antibody and Strep-Tactin Sepharose were purchased from IBA (Gottingen, Germany). Sepharose 6B and Glutathione 4B were obtained from GE Healthcare (Little Chalfont, UK). Human recombinant TGF-β1 and an anti-BrdU monoclonal antibody were purchased from Sigma (St. Louis, MO, USA). Dextran sulfate sodium (DSS; M.W. = 36–50 kDa) was obtained from MP Biomedicals (Santa Ana, CA, USA).

### Animals

Gadd45β-KO and C57BL/6 J mice (The Jackson Laboratory) were housed at a constant temperature (20–22 °C) on a 12:12-h light/dark schedule. All animal experiments were approved by the Institutional Animal Care and Use Committee of the Korea Research Institute of Bioscience and Biotechnology (KRIBB-AEC-16165) and conducted in accordance with the committee’s guidelines. Ten-week-old male mice were used for the experiments. Acute colitis was induced by administering 3% or 5% (w/v) DSS in the drinking water. For the repair experiment, mice were acclimatized to 3% DSS for 5 days and then provided regular drinking water for 3 or 5 days. Weight changes were calculated as the percent change in weight compared with the baseline weight, and macroscopic scoring of colon tissue was estimated according to the following grading system: 0 = no inflammation, 1 = swelling or redness, 2 = swelling and redness, 3 = one or two ulcers, 4 = more than two ulcers or one large ulcer, 5 = mild necrosis, and 6 = severe necrosis. Colons were dissected and washed with phosphate-buffered saline (PBS). The distal colon was fixed in 10% neutral buffered formalin (BBC Biochemical, Mt. Vernon, WA, USA), and the other portion was frozen in liquid nitrogen (LN2) and stored at −80 °C.

### Cell culture and transfection

Caco-2, HEK293T, and HeLa cells were cultured in Dulbecco’s Modified Eagle’s medium (DMEM; HyClone, Logan, UT, USA) containing 10% fetal bovine serum (FBS; HyClone) and 1% penicillin-streptomycin (Gibco, Grand Island, MA, USA). For overexpression of proteins, cells were transfected with DNA plasmid constructs using Lipofectamine 2000 (Invitrogen).

### Plasmid construction

DNA templates corresponding to the coding sequences of the human *Gadd45β* and *Smad7* genes were amplified by PCR. GST-tagged *Smad7* or truncated mutant constructs were cloned into the BamHI and NotI sites of the pEBG vector. Strep-tagged *Gadd45β* was cloned into the EcoRI and XhoI sites of the pEXPR-IBA105 vector (IBA). Flag-tagged *Smad7* was cloned into the AflII and XbaI sites of the pIRES vector. HA-tagged Smad7 was cloned into the BamHI and NotI sites of the pCMV vector. All constructs were sequenced by Bioneer Corporation (Daejeon, South Korea) to verify 100% correspondence with the original sequences. Myc-tagged or Flag-tagged Smurf1 was purchased from Addgene Corporation (Cambridge, MA, USA).

### RNA interference

Cells were transfected with siRNA using Lipofectamine RNAiMAX (Invitrogen) to downregulate Gadd45β expression according to the manufacturer’s protocol. A scrambled siRNA (Invitrogen) was used as the control. The following sequences specifically targeting human *Gadd45β* cDNA were used: 5′-ACGAGUCGGCCAAGUUGAUGAAUGU-3′, 5′-CAGUCCUUCUGCUGUGACAACGACA-3′, and 5′-GAGGUGGCCAGCUACUGCGAAGAAA-3′.

### In vitro proliferation assay

Caco-2 cells were plated in an eight-chamber slide 2 days before the experiment. Thirty-six hours after transfection with an empty vector or a V5-tagged Gadd45β plasmid, cells were treated with 10% DSS for 24 h. Thereafter, the medium was replenished with or without TGF-β (10 ng/ml) for 24 h to establish the recovery phase. The cells were washed with PBS twice, fixed with 4% paraformaldehyde (PFA) for 20 min, permeabilized with methanol for 20 min at −20 °C and then blocked with 2% BSA for 1 h. The cells were then incubated with an anti-Ki-67 antibody (0.1 μg/ml) overnight at 4 °C and incubated at 37 °C for 1 h with an Alexa 488-conjugated goat anti-rabbit IgG antibody (Invitrogen) for 1 h. Nuclear DNA was stained with DAPI. A Nikon laser-scanning confocal microscope (Nikon Corporation, Tokyo, Japan) was used to capture images, which were analyzed using NIS-Elements software (Nikon Corporation, Tokyo, Japan).

### GST and strep pull-down assays

At 36 h post transfection, cells were harvested and lysed in RIPA buffer (50 mM Tris-HCl, 150 mM NaCl, 1% NP40, 0.5% Na-deoxycholate, and 0.1% SDS) containing a protease inhibitor cocktail (Roche, Mannheim, Germany). The lysates were precleared with Sepharose 6B (GE Healthcare) for 1 h at 4 °C and incubated with Glutathione 4B (GE Healthcare) for GST pull-down or with Strep-Tactin Sepharose (IBA) for Strep pull-down overnight at 4 °C. Thereafter, the precipitated beads were washed three times with 1% NP40 and eluted with an SDS loading buffer by boiling for 10 min.

### Bone marrow transplantation

Bone marrow transplantation experiments were performed as described previously^17^. Briefly, male Gadd45β and C57BL/6 J mice were euthanized via cervical dislocation, and both femurs were dissected. The bone marrow was flushed using PBS, a single-cell suspension was prepared via gentle straining through a 70-μm Falcon^TM^ Cell Strainer (Life Sciences, Tewksbury, MA, USA), and 2.5 × 10^6^ cells were injected into recipient mice via the tail vein. Eight weeks after transplantation, the reconstituted Gadd45β and C57BL/6J mice were administered 3% DSS in the drinking water ad libitum for the indicated period. The success of bone marrow reconstitution was determined via PCR according to the genotyping protocol of The Jackson Laboratory (stock number 013101).

### Histological analysis and colonic injury scoring

A colon sample was serially sectioned at 5- and 100-µm thickness or prepared as Swiss rolls and stained with hematoxylin and eosin or periodic acid-Schiff (PAS). A pathologist blinded to group allocation assessed colitis severity. Colonic injury was assessed using a histological scoring system^18^. Briefly, the histological score was a combination of the scores for inflammation severity (0, normal; 1, mild; 2, moderate; and 3, severe), inflammation extent (0, none; 1, mucosa; 2, mucosa and submucosa; and 3, transmural), crypt damage (0, none; 1, basal 1/3 damaged; 2, basal 2/3 damaged; 3, crypts lost with surface epithelium present; and 4, crypts and surface epithelium lost), and percentage involvement (0, 0%; 1, 1–25%; 2, 26–50%; 3, 51–75%; and 4, 75–100%). The histological score was the sum of the scores for all parameters.

### In vivo proliferation assay

For BrdU-based evaluation, mice were injected with a solution (0.1 mg/g body weight) of BrdU (Sigma-Aldrich) diluted in PBS 2 h before euthanasia. Formalin-fixed, paraffin-embedded colon sections placed on coated slides were sequentially deparaffinized and rehydrated using xylene and ethanol. The slides were then treated for 15 min with boiling in a citrate buffer (20 mM sodium citrate, 0.05% Tween 20, pH 6.5). The slides were incubated for 1 h at room temperature in a normal blocking serum and then incubated overnight at 4 °C with an anti-BrdU primary antibody. The slides were washed with PBS, incubated with an Alexa Fluor 488-conjugated secondary antibody for 1 h at room temperature in the dark, rinsed with PBS, and mounted with an antifade reagent and DAPI. Images were obtained using a Carl Zeiss microscope (Jena, Germany). For PCNA staining, slides boiled in the citrate buffer were incubated for 1 h at room temperature in a normal blocking serum and incubated overnight at 4 °C with an anti-PCNA primary antibody. Next, the slides were washed with PBS, incubated with an Alexa Fluor 546-conjugated secondary antibody for 1 h at 37 °C, rinsed with PBS, and mounted with the antifade reagent and DAPI. Images were obtained using the Carl Zeiss microscope.

### Immunofluorescence staining

HeLa cells were seeded on an eight-chamber slide 1 day before the experiment. Thirty-six hours after transfection with a GST-tagged Smad7 plasmid, the cells were mock treated or treated with TGF-β (20 ng/mL) for 10 min. Thereafter, the cells were washed with PBS twice and fixed with 4% paraformaldehyde (PFA) for 20 min, followed by incubation with methanol for 20 min at −20 °C for permeabilization. The fixed cells were blocked with 2% FBS for 1 h and incubated with an anti-GST antibody (1:100) and anti-Gadd45β antibody (1:100) overnight at 4 °C. Thereafter, the cells were washed with PBST and incubated with Cy3-conjugated donkey anti-mouse IgG (The Jackson Laboratory) and Alexa 488-conjugated goat anti-rabbit IgG (Invitrogen) antibodies for 1 h at room temperature in the dark, blocking light exposure. After washing, the cells were incubated with 1 μg/mL DAPI (Sigma-Aldrich) with 0.01% RNase A and washed with PBST. Fluorescence images were captured using a Nikon laser-scanning confocal microscope and processed using NIS-Elements software.

### In vitro wound assays

To investigate the role of Gadd45β in wound healing, Gadd45β was silenced or overexpressed in Caco-2 cells with Gadd45β siRNA (Invitrogen) or V5-tagged Gadd45β plasmids, respectively. To determine the effect of Gadd45β ablation, Caco-2 cells were transfected with a scrambled siRNA or Gadd45β siRNA (20 nM), and confluent monolayer cells were wounded with a 100-μm tip and subsequently cultured in the absence or presence of TGF-β (10 ng/mL). Wound healing was observed and imaged at 0 and 48 h. To investigate the effects of Gadd45β overexpression, cells were transfected with an empty vector or V5-tagged Gadd45β plasmids (0.5 μg/ml) and cultured to confluence as a monolayer. A wound was induced using a 100-μL pipette tip (0.5-mm diameter) attached to a vacuum source, and suction was applied for ~1 s. Twenty-four hours before the wound assay, cells were treated with or without 2 mM hydroxyurea, a cell cycle blocker, and wounded cells were further incubated in medium with or without TGF-β (10 ng/mL) for 24 h, as reported previously^19^. Wound healing was observed and imaged at 0 and 24 h. The percentage of cell-covered area was calculated using the following equation:

$$\begin{array}{l}{\mathrm{Percentage}}\,{\mathrm{of}}\,{\mathrm{cell}}\hskip -2pt-\hskip -2pt{\mathrm{covered}}\,{\mathrm{area }}\\ = \left[ {\left( {{\mathrm{surface}}\,{\mathrm{area}}\left( {t = 0\,{\mathrm{h}}} \right)-{\mathrm{surface}}\,{\mathrm{area}}\;\left( {t = 24\,{\mathrm{h}}} \right)} \right)/{\mathrm{surface}}\,{\mathrm{area}}\;\left( {t = 0\,{\mathrm{h}}} \right)} \right]{\mathrm{\,x\,100}}\end{array}$$

### RNA isolation and qRT-PCR

Total RNA was isolated from colon tissue using TRIzol reagent (Invitrogen) per the manufacturer’s instructions. The RNA concentration was determined spectrophotometrically. Double-stranded cDNA synthesis was performed using the iScript^TM^ cDNA Synthesis Kit (BioRad, Hercules, CA, USA). The resulting cDNA was subjected to qPCR using a StepOnePlus^TM^ Real-Time PCR system (Applied Biosystems, Foster City, CA, USA) with AccuPower® 2 × Greenstar qPCR Master Mix (Bioneer, Daejeon, Korea) according to the standard protocol. A list of primer sequences is provided in supplementary Table. Gene expression of target genes was normalized to that of 18S rRNA.

### Immunoblot analysis

Colon tissue samples and cell lysates were homogenized in RIPA buffer with the Complete® Protease Inhibitor Cocktail (Roche Applied Science). Protein concentrations were quantified via the Bradford assay. Proteins were separated via electrophoresis on a 10–12% sodium dodecyl sulfate (SDS)-polyacrylamide gel, transferred to polyvinylidene fluoride (PVDF) membranes and probed with appropriate primary and secondary antibodies.

### Statistical analysis

The results of the immunoblot analysis were quantified using Tina2.0 software. Statistical analyses were performed using SPSS and Excel. Results are expressed as the mean ± SEM of three independent experiments. Between-group comparisons were performed using a two-tailed Student’s *t* test, multiple-group comparisons were performed by one-way ANOVA (Tukey), and survival differences between groups were analyzed by the log-rank (Mantel-Cox) test. A *P*-value < 0.05 was considered statistically significant.

## Results

### Gadd45β ablation causes hypersusceptibility to experimental colitis

**Fig. 1:**
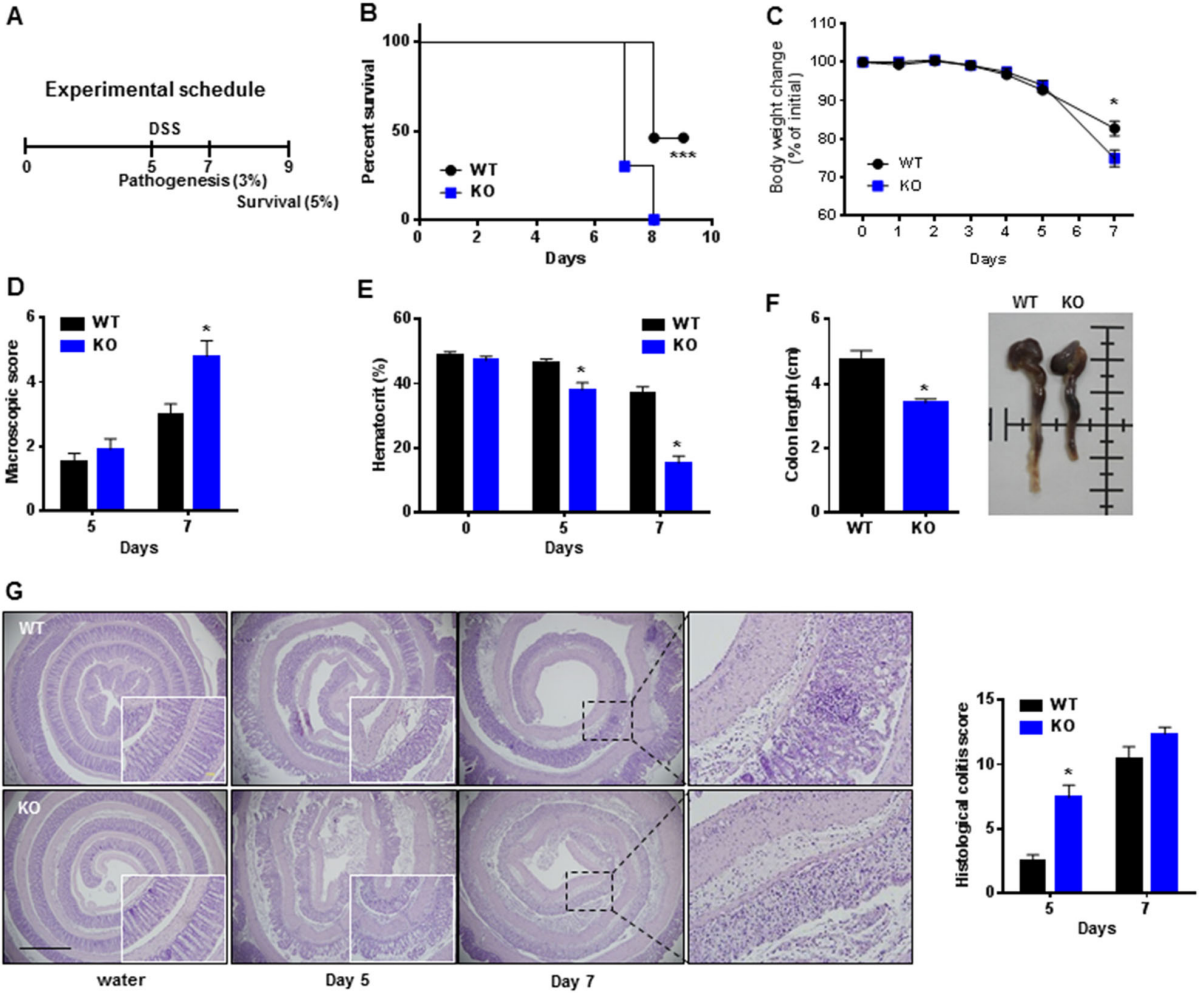
Increased susceptibility of Gadd45β-KO mice to dextran sulfate sodium (DSS)-induced colitis. **a** A schematic representation of the experimental design is shown. **b** The survival rates of Gadd45β-WT (*n* = 13, black circle) and Gadd45β-KO (*n* = 10, blue square) mice after treatment with 5% DSS are presented as percentages, and survival differences between the groups were analyzed via the log-rank (Mantel-Cox) test. ***P *<* 0.001. **c** The body weights of Gadd45β-WT (*n* = 5) and Gadd45β-KO (*n* = 5) mice after treatment with 3% DSS are presented as percentages of the starting weight of each mouse. Statistical significance was determined via Student’s *t* test. *P *<* 0.05. **d**–**f** Macroscopic scores, hematocrit percentage, and colon length were estimated for Gadd45β-WT (*n* = 5) and Gadd45β-KO (*n* = 5) mice at 5 or 7 days after treatment with 3% DSS. Statistical significance was determined via Student’s *t* test. *P *<* 0.05. **g** Representative hematoxylin and eosin staining (left) and histological scores (right) of the colon of Gadd45β-WT (*n* = 6) and Gadd45β-KO (*n* = 6) mice are shown. The bar represents 1 mm. Statistical significance was determined via Student’s *t*-test. *P *<* 0.05

We evaluated Gadd45β expression in several tissues from C57BL/6 mice via quantitative real-time PCR and immunoblotting. The mRNA and protein levels of Gadd45β were markedly upregulated in the colon and ileum but downregulated in the brain, kidneys, and liver (Supplementary Fig. 1a, b). Gadd45β-positive cells were more frequent in the colon and ileum than in the kidneys, and liver and epithelial cells were prominently stained, suggesting a possible role for Gadd45β in the intestine (Supplementary Fig. 1c). Under normal conditions, Gadd45β-KO mice had a healthy normal intestine, as evidenced by the intact expression of genes related to tight junction regulation (Supplementary Fig. 2a). Then, Gadd45β-KO and WT mice were challenged via oral administration of 5% DSS, and survival was monitored for 9 days (Fig. 1a). The Gadd45β-KO mice died at an earlier time point (70% death at 7 days) than the control mice (0% at 7 days) (Fig. 1b). In subsequent experiments, we used 3% DSS due to the high mortality rate observed with 5% DSS (Fig. 1a). Interestingly, DSS administration significantly upregulated Gadd45β expression, which peaked at 3 days in colon tissues (Supplementary Fig. 2b). Gadd45β-KO mice displayed greater weight loss than WT mice (Fig. 1c). Macroscopic scores were higher for Gadd45β-KO mice than for WT mice at 7 days after DSS (Fig. 1d). Hematocrit percentage (Fig. 1e) and colon length (Fig. 1f) were significantly decreased in Gadd45β-KO mice compared to WT mice. Histological analyses clearly revealed larger areas of ulceration and crypt loss and more pronounced submucosal inflammatory cell infiltration in Gadd45β-KO mice than in WT mice (Fig. 1g). These data suggest that Gadd45β plays a protective role in colitis pathogenesis in vivo. We evaluated the mRNA levels of other Gadd45 molecules (*Gadd45α* and *Gadd45γ*) in the colon of Gadd45β-WT and Gadd45β-KO mice to determine the presence of compensatory effects within the family; however, no significant differences in *Gadd45α* or *Gadd45γ* expression were observed (Supplementary Fig. 2c). UC is characterized by an excessive response of the intestinal immune system and marked upregulation of proinflammatory cytokine levels^20^. Therefore, we compared cytokine expression in colon tissues from WT and those from Gadd45β-KO mice at 5 and 7 days after DSS administration. *IL-1β*, *IL-6*, and *IL-10* mRNA levels were similarly drastically upregulated in the colons from Gadd45β-WT and Gadd45β-KO mice after DSS treatment (Supplementary Fig. 3). We further analyzed other signaling pathways involved in cell survival and apoptosis, such as signal transducer and activator of transcription 3 (STAT3) and protein kinase B (PKB, Akt) pathways^21,22^. In DSS-induced colitis, STAT3 phosphorylation was significantly increased in the colon tissue of Gadd45β-KO mice compared to that of WT mice, while active PKB phosphorylation in colon tissue was not altered by Gadd45β (Supplementary Fig. 4).

### Gadd45β plays a prominent role in the epithelium in DSS-induced colitis

**Fig. 2:**
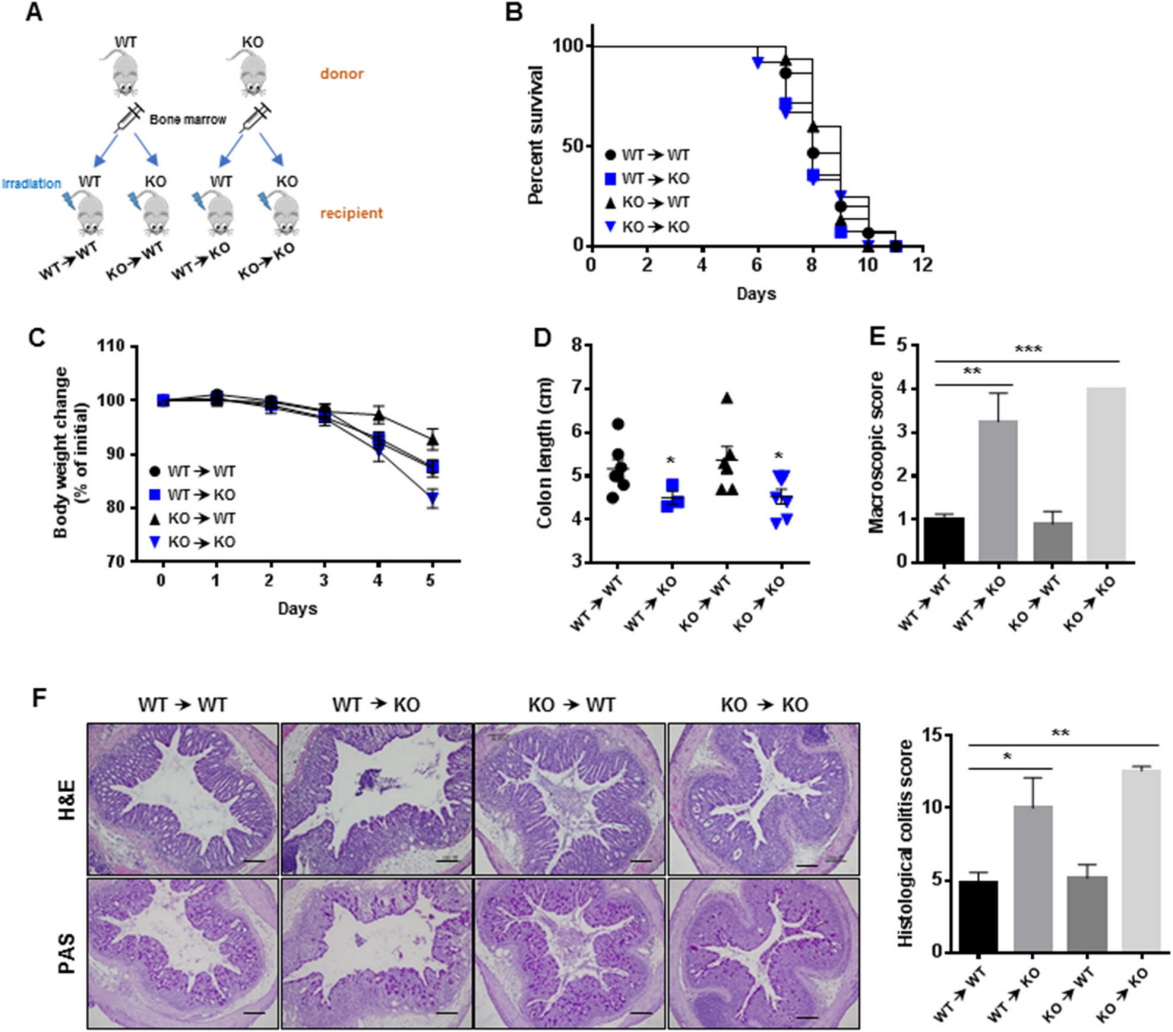
Increased response to DSS-induced colitis in Gadd45β-KO mice is dependent on the epithelium of the colon. **a** A schematic representation of the experimental design is shown. **b** The survival rates in the WT → WT (*n* = 15, black circle), WT → KO (*n* = 14, blue square), KO → WT (*n* = 15, black triangle), and KO → KO (*n* = 12, blue inverted triangle) groups were monitored during the experimental period after treatment with 3% DSS. **c**–**e** Body weights, changes in colon length, and macroscopic scores were measured in the WT → WT (*n* = 6), WT → KO (*n* = 3), KO → WT (*n* = 6), and KO → KO (*n* = 6) groups at 5 days after treatment with 3% DSS. Statistical significance was determined via one-way ANOVA (Tukey). *P *<* 0.05, **P *<* 0.01 and ***P *<* 0.001. **f** Representative images of colonic tissue stained with hematoxylin and eosin (H&E; upper) or periodic acid-Schiff’s reagent (lower) are shown. The bar represents 200 μm. Histological colitis scores were estimated for each H&E-stained colon section in each group (*n* = 6 per group). Statistical significance was determined via one-way ANOVA (Tukey). *P *<* 0.05 and **P *<* 0.01. KO → WT indicates that a WT mouse received KO bone marrow

To investigate the precise roles of hematopoietic and epithelial cells in experimental colitis, we generated chimeric mice using irradiated Gadd45β-WT and Gadd45β-KO recipient mice reconstituted with bone marrow cells from Gadd45β-WT or Gadd45β-KO donors and challenged these mice with DSS at 8 weeks posttransplantation (Fig. 2a). The degree of chimerism was estimated via PCR using blood samples (Supplementary Fig. 5). The Gadd45β-KO recipient mice transplanted with Gadd45β-WT or Gadd45β-KO bone marrow displayed lower survival rates than the Gadd45β-WT recipient mice transplanted with bone marrow cells from Gadd45β-WT or Gadd45β-KO mice (Fig. 2b). However, we only observed slight body weight differences between the Gadd45β-KO mice transplanted with Gadd45β-KO bone marrow cells and the Gadd45β-WT mice transplanted with Gadd45β-WT bone marrow cells (Fig. 2c). Colon length was reduced, and macroscopic scores were higher in the Gadd45β-KO mice transplanted with Gadd45β-WT or Gadd45β-KO bone marrow cells than in the Gadd45β-WT recipient mice (Fig. 2d, e). Furthermore, a higher histological score and severe loss of goblet cells were observed in the colon of the Gadd45β-KO recipient mice regardless of donor cell type (Fig. 2f). We compared cytokine expression in the colon tissue samples of each group, but no significant differences were observed (Supplementary Fig. 6). These data suggest that Gadd45β plays a pivotal role in epithelial cells during colitis progression.

### Gadd45β enhances the proliferation of intestinal epithelial cells in a TGF-β-independent manner in vitro

**Fig. 3:**
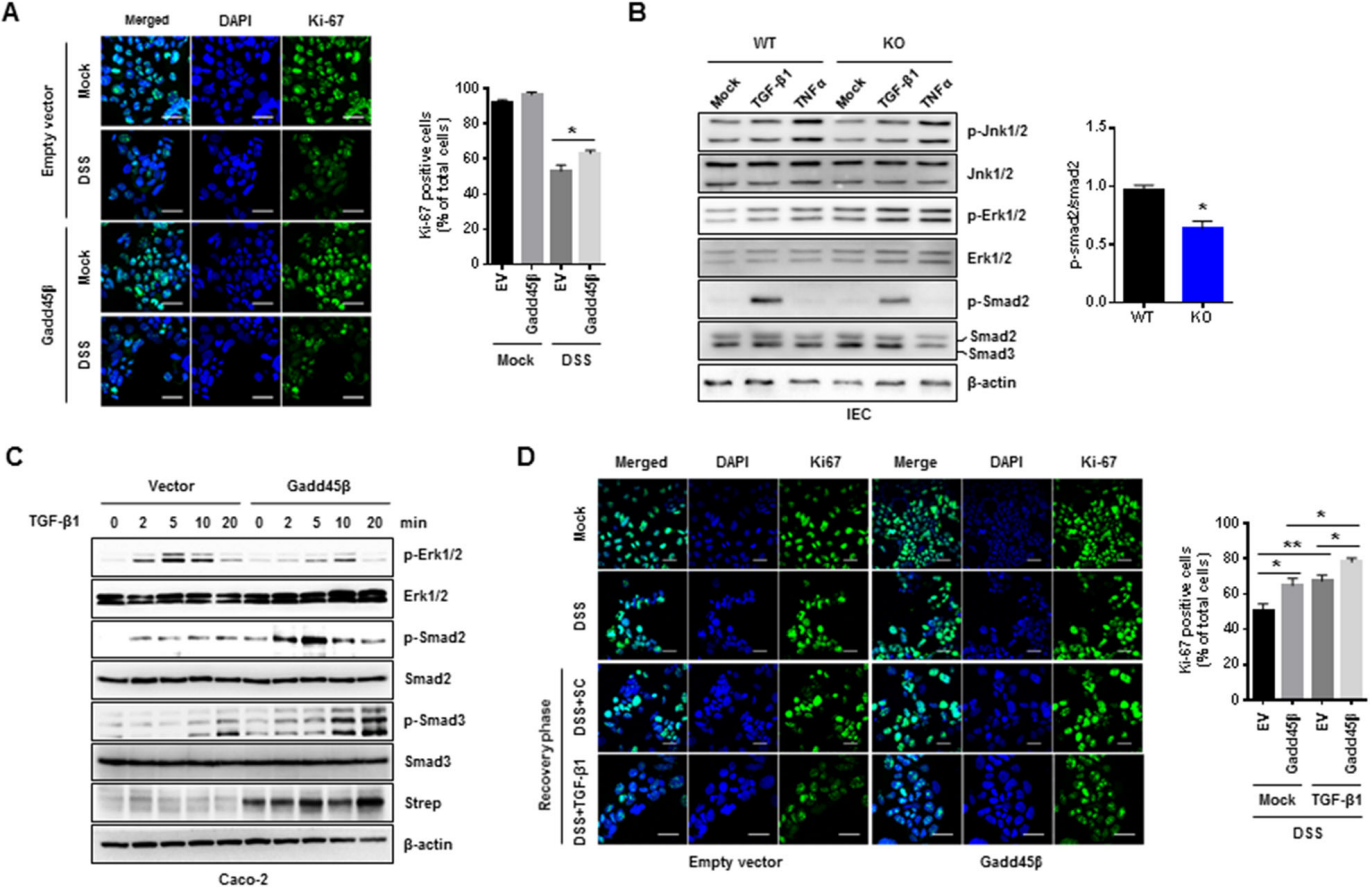
Gadd45β regulates the TGF-β signaling pathway in vitro. **a** Caco-2 cells were transfected with Gadd45β or an empty vector and cultured for 36 h. After 36 h of culturing, the cells were treated with/without DSS for 24 h, fixed and stained with an anti-Ki-67 antibody and DAPI. The percentage of Ki-67-positive cells was determined by analyzing more than 10 fields. Data are representative of at least two independent experiments. *P *<* 0.05. **b** Intestinal epithelial cells isolated from whole colons of Gadd45β-WT or Gadd45β-KO mice were treated with DMSO, human TGF-β1 (10 ng/mL) or human TNF-α (50 ng/mL) for 1 h, and cell lysates were used for immunoblotting using specific antibodies against the proteins indicated in the figure. Statistical significance was determined via Student’s *t* test. *P *<* 0.05. **c** Caco-2 cells overexpressing the empty vector or Gadd45β were treated with 10 ng/mL TGF-β1, and cell lysates were used for immunoblotting using specific antibodies against the proteins indicated in the figure. **d** After transfection with the empty vector or Gadd45β, Caco-2 cells were treated with 10% DSS for 24 h and further incubated in fresh complete medium with/without TGF-β (10 ng/mL) for 24 h. Proliferating cells were identified via Ki-67 staining, and the percentage of Ki-67-positive cells was determined by analyzing more than 10 fields. Data are representative of at least two independent experiments. Statistical significance was determined via Student’s *t* test. *P *<* 0.05 and **P *<* 0.01

We further assessed the physiological roles of Gadd45β in DSS-induced apoptosis in Caco2 cells, a human epithelial cell line. Caco-2 cells overexpressing Gadd45β were more resistant to DSS-induced apoptosis than were control cells (Fig. 3a). Several signaling pathways contribute to IBD pathogenesis^23–25^. However, the cellular mechanism by which Gadd45β mediates DSS-induced colitis is unclear. Therefore, we analyzed signaling pathways responding to TGF-β or TNFα in isolated primary intestinal epithelial cells (IECs) and found reduced responses to TGF-β in Gadd45β-depleted IECs compared with Gadd45β-WT IECs (Fig. 3b). In contrast, Gadd45β upregulation in Caco-2 cells enhanced the phosphorylation of molecules downstream of TGF-β, including Smad2 and Smad3 (Fig. 3c). We further assessed the roles of the Gadd45β-regulated TGF-β signaling pathway in proliferation during recovery. Caco-2 cells overexpressing an empty vector or Gadd45β were stimulated with DSS for 24 h and allowed to recover in fresh medium with or without TGF-β for 24 h. The Caco-2 cells overexpressing Gadd45β displayed significantly increased proliferation compared to the control cells, and TGF-β treatment further enhanced proliferation during the recovery phase (Fig. 3d). However, the increased rates of cellular proliferation in response to TGF-β were similar in both treatment groups, suggesting that the Gadd45β-induced TGF-β signaling pathway is insufficient to regulate cellular proliferation during recovery after DSS elimination.

### Gadd45β-mediated TGF-β signaling pathway promotes the progression of epithelial restitution in vitro

**Fig. 4:**
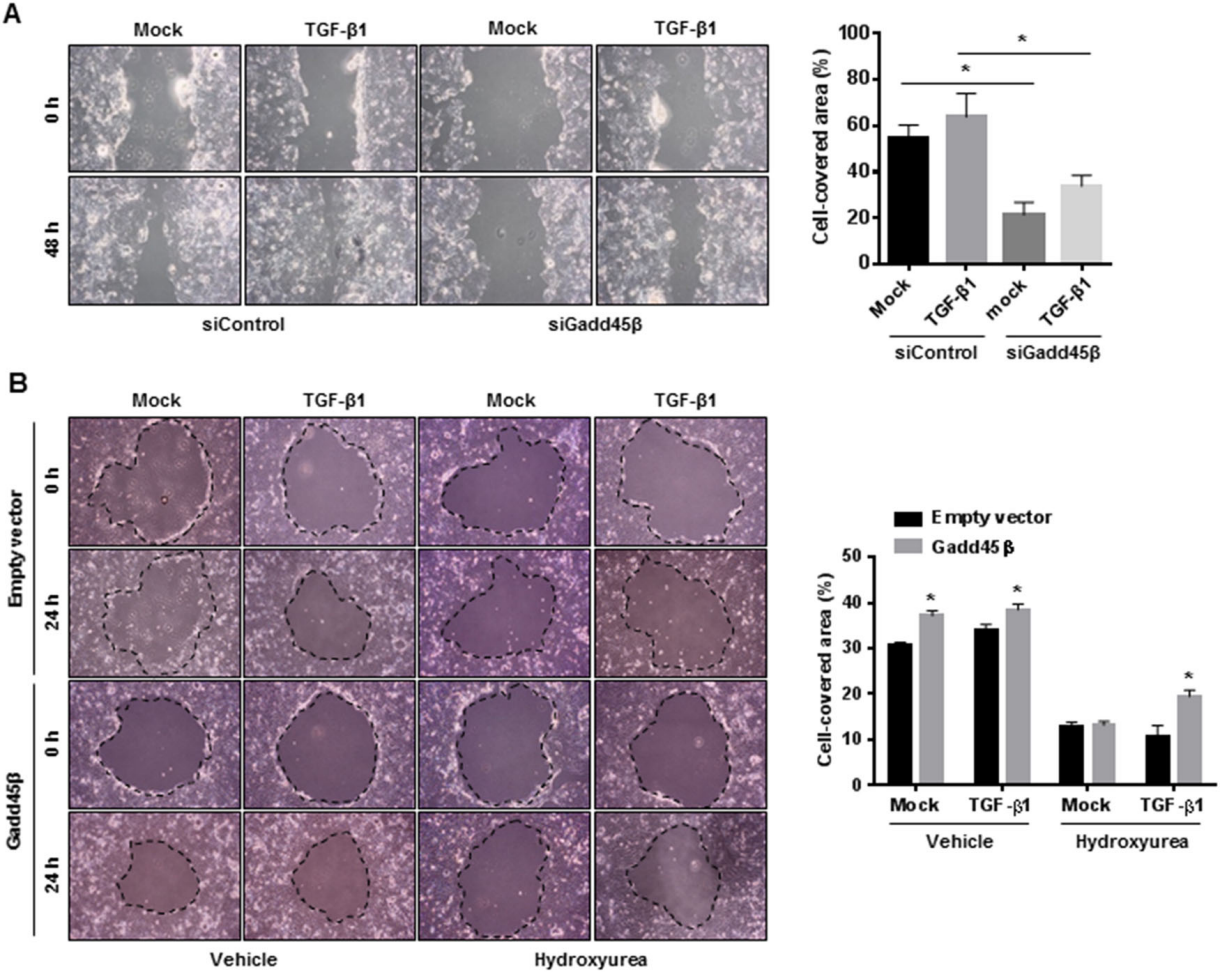
Involvement of the Gadd45β-mediated TGF-β signaling pathway in epithelial restitution in vitro. **a** A wound healing assay was performed using Caco-2 cells transfected with a scrambled siRNA (siControl) or Gadd45β-specific siRNA in the absence or presence of TGF-β1 (10 ng/mL). The cell-covered area is presented as the percentage of the wound healing area. Measurements of the wound surface area were performed. **b** Caco-2 cells overexpressing an empty vector or Gadd45β were cultured to confluence as a monolayer. Twenty-four hours before the wound healing assay, the cells were treated with or without 2 mM hydroxyurea, and wounded monolayers were further incubated in medium with or without TGF-β1 (10 ng/mL) for 24 h. Measurements of the wound surface area were carried out. The results are expressed as the mean ± SEM. Statistical significance was determined via Student’s *t* test. *P *<* 0.05

TGF-β enhances wound healing in epithelial cells^26^. To determine the role of Gadd45β-induced TGF-β signals in wound healing, Caco-2 cells were treated with a scrambled siRNA or Gadd45β-specific siRNA, which reduced mRNA levels by 50% compared to the scrambled siRNA (Supplementary Fig. 7). Although the Gadd45β-specific siRNA significantly decreased wound healing activity compared with the control siRNA, the wound healing response rate to TGF-β in the Gadd45β-silenced cells did not differ from that in the control cells (Fig. 4a). We investigated the effect of Gadd45β overexpression on wound healing in Caco-2 cells. In contrast to the silencing data, the data from this experiment showed that Gadd45β upregulation significantly enhanced wound healing compared to control expression, as determined by measuring the cell-covered area (Fig. 4b). TGF-β treatment slightly induced wound closure in both cells overexpressing Gadd45β and those expressing an empty vector to a similar extent (Fig. 4b). We next assessed TGF-β-mediated restitution independent of cell proliferation. Caco-2 cells expressing Gadd45β or an empty vector were pretreated with hydroxyurea for 24 h to block cell proliferation, which helped evaluate the role of cell migration in epithelial restitution. Interestingly, the wound closure response to TGF-β was significantly enhanced in the cells overexpressing Gadd45β with inhibited cell proliferation upon hydroxyurea treatment compared to the cells overexpressing the empty vector, while hydroxyurea alone completely inhibited Gadd45β-mediated wound healing, suggesting that TGF-β signaling is involved in Gadd45β-induced restitution by stimulating cell migration (Fig. 4b).

### TGF-β signaling pathways are regulated by inhibiting the Smurf-mediated degradation of a TGF-β receptor via competitive binding of Gadd45β to Smad7

**Fig. 5:**
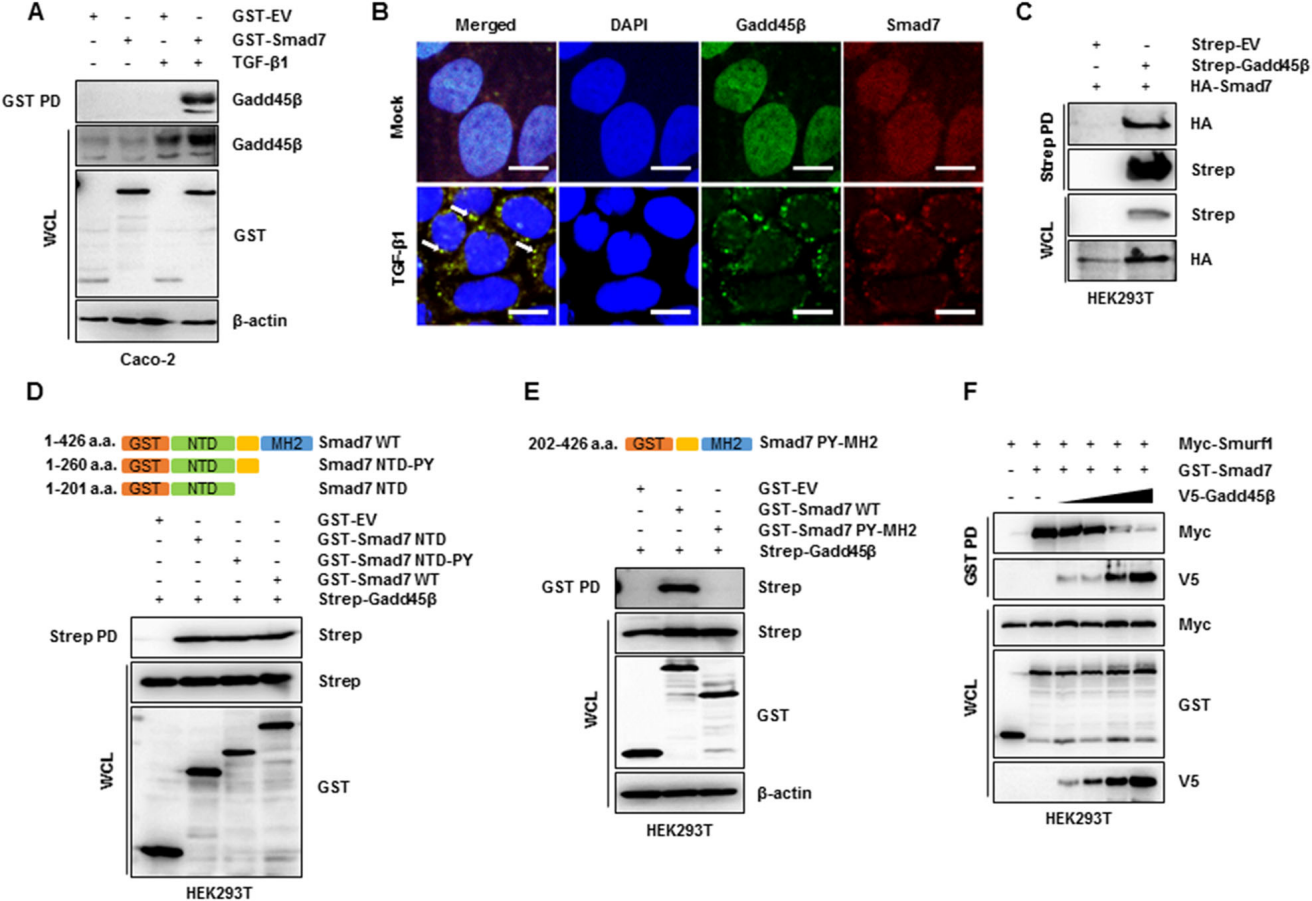
Interruption of the Smad7/Smurf1 complex via direct binding of Gadd45β to the Smad7 N-terminal domain. **a** GST-empty vector- and GST-tagged Smad7-overexpressing Caco-2 cells were treated with or without TGF-β1 (10 ng/mL) for 10 min, and cell lysates were used for a GST pull-down assay, followed by immunoblotting with an anti-Gadd45β antibody. Whole-cell lysates were used to evaluate Gadd45β expression, and β-actin was used as the control. **b** At 36 h post transfection with GST-tagged Smad7, HeLa cells were mock treated or treated with TGF-β (10 ng/mL) for 10 min and evaluated via confocal microscopy analysis of staining with anti-GST and anti-Gadd45β antibodies together with fluorophore-conjugated secondary antibodies. The bar represents 20 μm. The white arrows indicate colocalization of Gadd45β (green) and Smad7 (red). **c** HEK293T cells were transfected with the indicated plasmids, and cell lysates were used for a Strep pull-down assay, followed by immunoblotting with an anti-HA antibody and an anti-Strep antibody. Whole-cell lysates were subjected to immunoblotting using the indicated antibodies. (**d**, upper) A diagrammatic representation of GST-tagged Smad7 (WT or mutant) constructs is shown. (**d**, lower) HEK293T cells were transfected with the GST-empty vector or GST-tagged Smad7 mutants together with Strep-tagged Gadd45β, and cell lysates were used for a Strep pull-down assay, followed by immunoblotting with an anti-Strep antibody. Whole-cell lysates were subjected to immunoblotting with the indicated antibodies. (**e**, upper) A diagrammatic representation of GST-tagged Smad7 (WT or mutant) constructs is shown. (**e**, lower) HEK293T cells were transfected with the GST-empty vector or GST-tagged Smad7 mutants together with Strep-tagged Gadd45β, and cell lysates were used for a GST pull-down assay, followed by immunoblotting with an anti-Strep antibody. Whole-cell lysates were subjected to immunoblotting with the indicated antibodies. **f** HEK293T cells were transfected with the GST-empty vector or GST-tagged Smad7 together with Myc-tagged Smurf1 and V5-tagged Gadd45β (dose dependent), and cell lysates were used for a GST pull-down assay, followed by immunoblotting with an anti-Myc or anti-V5 antibody

Among the regulatory Smads (R-Smads), Smad2, and Smad3 are substrates of TGF-β receptor type 1 (TβR1); they can be phosphorylated, bind to Smad4 and accumulate in the nucleus where they regulate transcription^27^. We first attempted to confirm the interaction between Gadd45β and Smad2 to understand the direct regulation of TGF-β signaling. However, Gadd45β did not bind to Smad2, despite TGF-β stimulation (Supplementary Fig. 8a, b). Interestingly, Gadd45β directly associated with Smad7, an inhibitory Smad (I-Smad) that forms a heterodimeric complex with the E3 ligase Smurf1^28,29^ (Fig. 5a). To further define the subcellular distributions of both proteins in the absence or presence of TGF-β, HeLa cells were transfected with GST-tagged Smad7. Smad7 typically accumulates in the nucleus; however, various stimuli, including TGF-β signaling, lead to cytoplasmic translocation from the nucleus^30^. In agreement with previous reports, our results show that the majority of Smad7 is located in the nucleus in the absence of TGF-β, and the cytosolic Smad7 level is upregulated significantly upon TGF-β treatment (Fig. 5b). Gadd45β was observed in both the nucleus and cytoplasm but was dominantly distributed in the cytosol after TGF-β treatment. The physical interaction between Smad7 and Gadd45β occurred mostly in the cytoplasm, not the nucleus. This interaction was strongly enhanced by TGF-β stimulation (Fig. 5b). To identify the Gadd45β-interacting domain of Smad7, HEK 293 cells were transfected with an HA-tagged Smad7 plasmid together with a Strep-tagged Gadd45β plasmid. Consistent with the Caco-2 data, the whole form of the Gadd45β protein was strongly associated with Smad7 in the HEK 293 cells (Fig. 5c). Smad7 comprises an N-terminal domain (NTD), an intervening linker region containing a PY motif and an MH2 domain. We generated constructs of GST-tagged Smad7 mutants (NTD-PY and NTD) or WT Smad7 and performed a GST pull-down assay. Gadd45β exhibited strong binding with the NTD of Smad7; however, NTD-deleted Smad7 did not bind to Gadd45β (Fig. 5d, e). Thus, we hypothesized that Gadd45β competitively interrupts mechanical interactions between Smad7 and Smurf. As expected, dose-dependent Gadd45β upregulation disrupted the Smad7/Smurf1 interaction (Fig. 5f). Taken together, the results indicated that TβR1 stability increased upon Gadd45β overexpression (Supplementary Fig. 8c), suggesting that Gadd45β essentially associates with the NTD of Smad7 and competes with Smurf1, thus stabilizing TβR1.

### Gadd45β-KO mice display impaired recovery of colon tissue from DSS-induced colitis

**Fig. 6:**
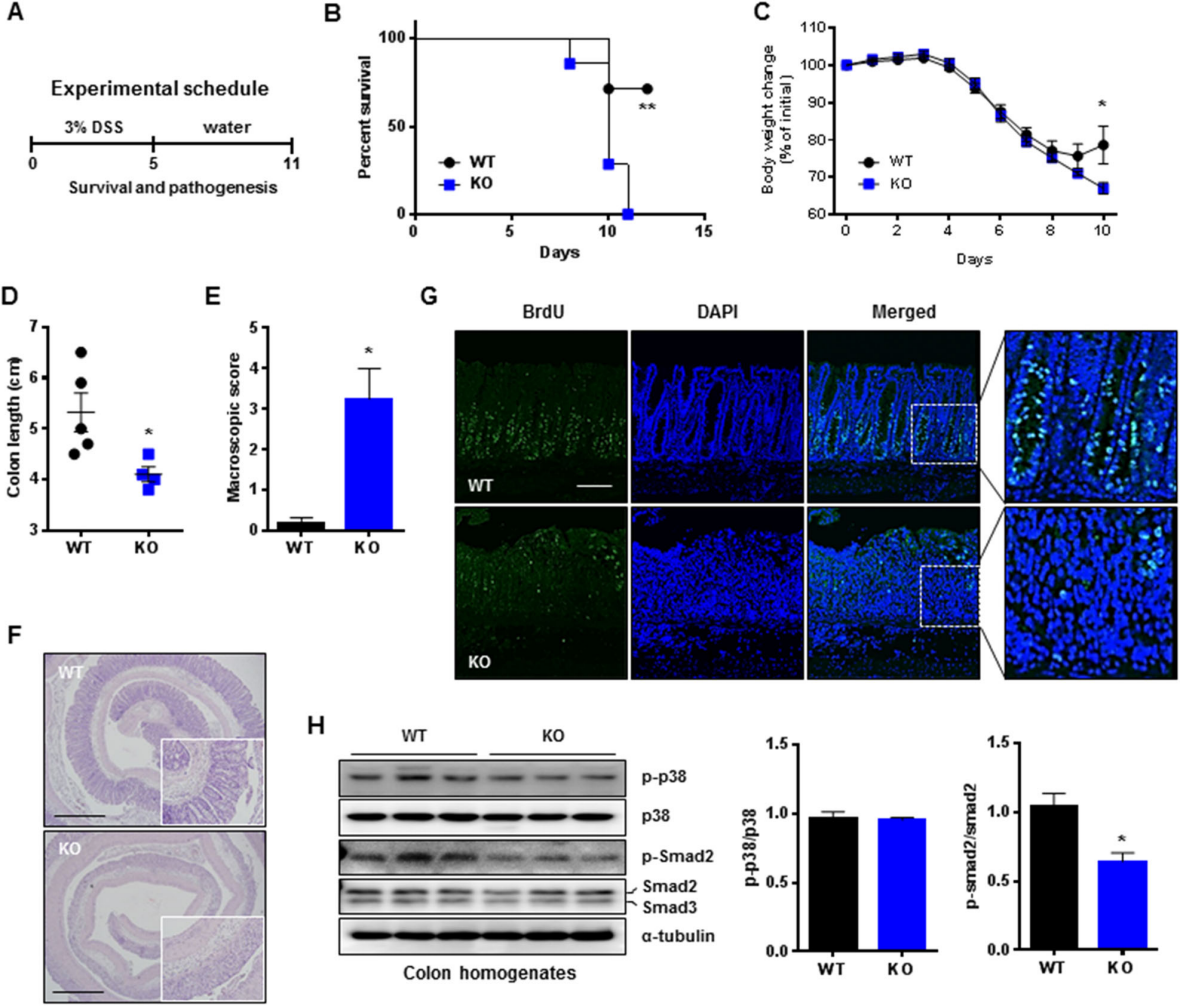
Delayed proliferation and disrupted repair of colon tissues in Gadd45β-KO mice during the recovery phase of ulcerative colitis. **a** Gadd45β-WT and Gadd45β-KO mice received 3% DSS in their drinking water for 5 days and were then permitted to recover by replacing the DSS with regular drinking water. **b** The survival rates of Gadd45β-WT (*n* = 7, black circle) and Gadd45β-KO (*n* = 7, blue square) mice after treatment with 3% DSS are presented as percentages. Differences in survival between the groups were analyzed via the log-rank (Mantel-Cox) test. **P *<* 0.01. **c**–**e** Body weight changes, colon lengths, and macroscopic scores were determined for Gadd45β-WT (*n* = 5–7) and Gadd45β-KO (*n* = 4–7) mice treated with 3% DSS for 10 days. Statistical significance was determined via Student’s *t* test. *P *<* 0.05. **f** Representative images of colons from Gadd45β-WT and Gadd45β-KO mice at day 3 of recovery stained with hematoxylin and eosin (left) are shown. The bar represents 1 mm. **g** Representative immunofluorescence images of colons from Gadd45β-WT and Gadd45β-KO mice at day 3 of recovery were obtained after staining with an anti-BrdU antibody (green) and DAPI (blue). The bar represents 200 μm. **h** Colon lysates from Gadd45β-WT and Gadd45β-KO mice at 3 days of recovery were used for immunoblotting, and the relative intensities of bands for the phosphorylated forms of each protein were quantified. Statistical significance was determined via Student’s *t* test. *P *<* 0.05

TGF-β plays a critical role in regenerating intestinal epithelial cells in DSS-induced colitis in mice^26^. We examined the repair of injured colons in WT and Gadd45β-KO mice after treatment with 3% DSS for 5 days and recovery for 5 days (DSS replaced with water during recovery period) (Fig. 6a). The survival of the Gadd45β-KO mice decreased significantly compared to that of the WT mice (Fig. 6b). The body weights of the WT mice after DSS drinking decreased rapidly for 7 days and increased slightly at 10 days, but body weight continued to decline rapidly at 10 days in the Gadd45β-KO mice (Fig. 6c). The Gadd45β-KO mice displayed significantly reduced colon lengths (Fig. 6d). Macroscopic scores were higher in the KO mice than in the WT mice at 10 days (Fig. 6e). Remarkably, the colonic epithelium of the WT mice exhibited a normal structure, while that of the Gadd45β-KO mice displayed severely disrupted epithelial integrity (Fig. 6f). Epithelial cell proliferation was evaluated via bromodeoxyuridine (BrdU) incorporation and proliferating cell nuclear antigen (PCNA) staining (Fig. 6g, Supplementary Fig. 9). Proliferation was prominent in the colonic crypts of the WT mice but not in those of the Gadd45β-KO mice, indicating that Gadd45β contributes to cell proliferation during repair of injured tissue. We further analyzed the phosphorylation of several proteins at 3 days of recovery. The active form of p38 in the colon was not significantly different between the mouse strains, but Smad2 activity was significantly decreased in the Gadd45β-KO mice compared to the WT mice (Fig. 6h). These results suggest that the TGF-β signaling pathway potentially contributes to the Gadd45β-mediated restoration of damaged colon tissue during recovery.

## Discussion

Gadd45β has been implicated in many biological processes, including DNA repair, cell cycle arrest, inflammation, cell survival, and apoptosis, depending on cell type^9–12^. These biological processes are closely involved in IBD pathogenesis^31^. Although the physiological roles of Gadd45β have been demonstrated in several cell types, the precise roles of Gadd45β in UC are unclear. This study shows a multifaceted role for Gadd45β in DSS-induced colitis.

Gadd45β is a small protein that lacks enzymatic activity and functions predominantly by interacting with partner proteins^9,10^. These interactions are regulated through the expression levels, cellular localization, and posttranslational modifications of both Gadd45β and its interacting partners^9^. Gadd45β mRNA and protein levels are upregulated in the small and large intestine rather than other tissues, suggesting their potential roles in the intestine. We assessed whether Gadd45β is involved in UC by using a mouse model of DSS-induced colitis, a well-defined chemical-induced model of colitis resembling human UC^32^. DSS initially damages the epithelial layer and activates innate immune responses^33^. Under these conditions, Gadd45β-KO mice developed more severe colitis than WT mice, as evidenced by the development of key clinical features of colitis (mortality, body weight loss, bloody diarrhea, and hematocrit changes). Consistent with these clinical signs, Gadd45β-KO mice also displayed notable histological alterations compared with control mice. These results indicate that Gadd45β has a protective role in DSS-induced colitis.

Gadd45β has been implicated in the vertebrate immune system, protecting hosts against pathogens^15^. Intercellular communication in the immune system is mediated by cytokines, which bind specific cell-surface receptors and activate intracellular signaling networks^34^. Gadd45β is an important component of intracellular signaling networks and is induced by numerous cytokines and bacterial LPS^10,11,13^. Furthermore, Gadd45β influences cytokine production and T cell differentiation^16^. Herein, concurrently, *Gadd45β* expression was upregulated in isolated intestinal epithelial cells upon treatment with several cytokines and LPS. However, inflammatory cytokine production in the colon did not differ between Gadd45β-KO and WT mice despite a marked increase in the inflammatory region in the colon of Gadd45β-KO mice, suggesting that Gadd45β might downregulate cytokine levels in the colon and that Gadd45β may regulate cytokine production in a cell type-dependent manner. We recently reported that the release of proinflammatory cytokines into the blood was decreased in Gadd45β-KO mice but increased in the lungs in experimental sepsis models^35^.

We further investigated the precise roles of hematopoietic and epithelial cells in Gadd45β-KO mice with exacerbated experimental colitis via bone marrow cell transplantation, revealing that Gadd45β-WT mice transplanted with bone marrow from WT or Gadd45β-KO animals exhibited similar colitis severity, which was evident through microscopic and histological analyses. Gadd45β-KO mice exhibited no improvement upon bone marrow transplantation from WT mice. Therefore, under stress conditions, including DSS treatment, UC severity in Gadd45β-KO mice primarily results from intestinal epithelial cell dysfunction.

Herein, we first identified Gadd45β as a novel TGF signaling pathway regulator. TGF-β is a key regulator of the intestinal epithelium and an immunosuppressive cytokine produced by many cell types^36^. Smads are proteins downstream of TGF-βR and regulate specific TGF-β target genes, thus constituting the canonical Smad pathway^37^. Furthermore, Smad3, an intracellular signaling protein in the canonical TGF-β pathway, is associated with IBD susceptibility^38^. Herein, Gadd45β ablation diminished active phosphorylation of Smad2 in response to TGF-β in vitro and in vivo. Conversely, Gadd45β-overexpressing cells exhibited more rapid Smad2/3 phosphorylation than control cells. TGF-β exerts a negative feedback effect that occurs in a Smad7-dependent manner^39^. We found that Gadd45β inhibited Smad7 by competing with Smurf via direct binding to Smad7. Smurf is an E3 ubiquitin-protein ligase that promotes TGF-βR proteasomal degradation^40^. In contrast, Gadd45β overexpression suppressed Smurf activity, suggesting that Gadd45β positively regulates the TGF-β signaling pathway in the intestinal epithelium. TGF-β is involved in intestinal epithelial homeostasis and is particularly associated with mucosal integrity and wound healing^41,42^. Concordantly, we show that Gadd45β-mediated TGF-β signaling is involved in epithelial restitution by enhancing epithelial cell migration across a wound but not epithelial cell proliferation. Consistent with in vitro data, in vivo data showed that the difference in DSS-induced colitis between Gadd45β-KO and Gadd45β-WT mice was more prominent during recovery than during colitis pathogenesis, suggesting important roles for Gadd45β in the repair of the injured intestinal epithelium during recovery. However, the roles of Gadd45β in immune cells during colitis remain unknown and must be further studied in the future.

**Fig. 7:**
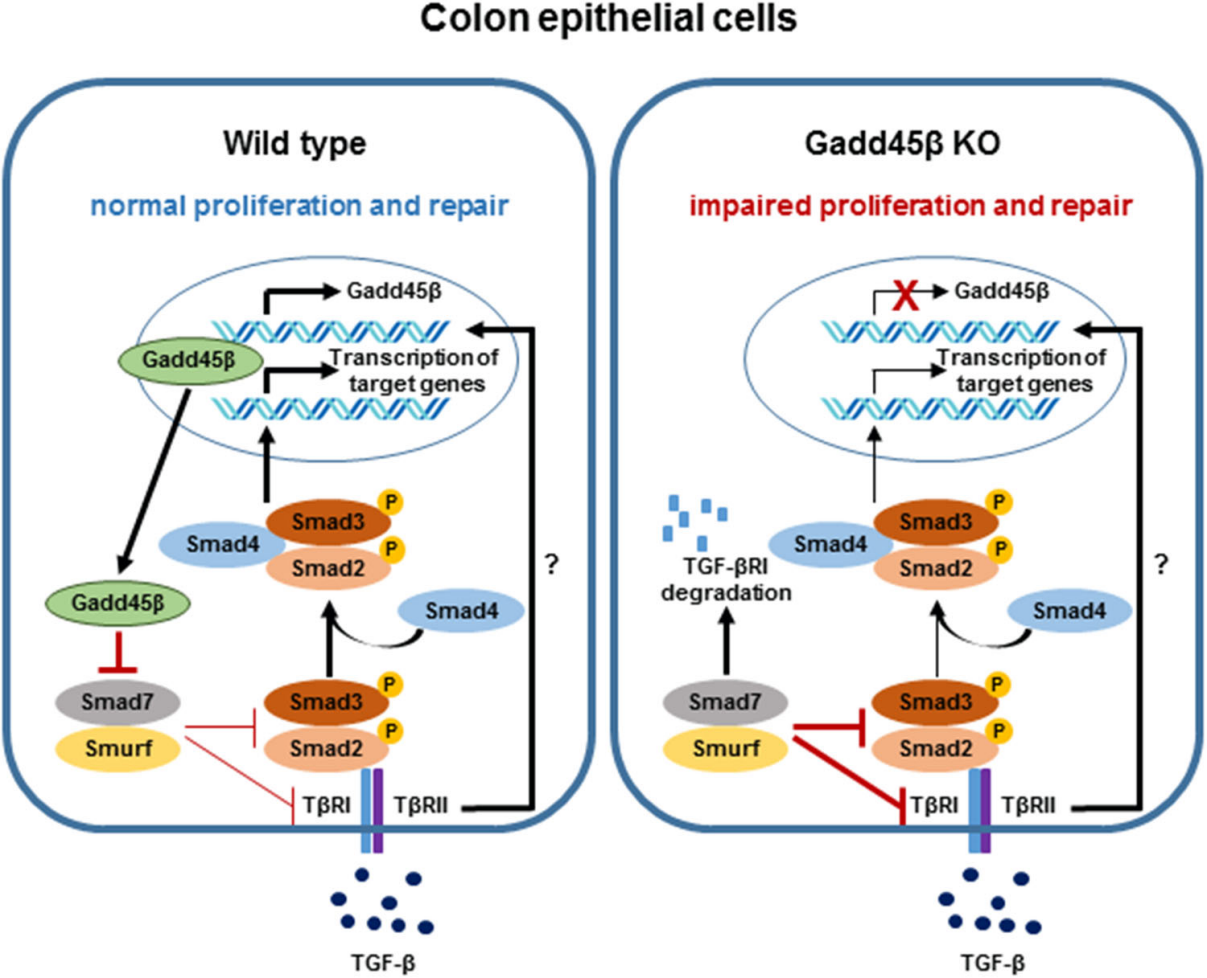
A schematic illustration of the roles of Gadd45β in intestinal epithelial cells (IECs). In Gadd45β-positive IECs, Gadd45β inhibits the SMAD7/Smurf complex and improves the stability of TGF-βRI during inflammatory bowel disease-related stress (left). However, in Gadd45β-negative IECs, the SMAD7/Smurf complex is enhanced, and the degradation of TGF-βRI is increased, resulting in abnormal TGF-β signaling and impaired proliferation and wound healing in the injured colon (right)

Overall, our results provide the first evidence supporting a role for Gadd45β in intestinal hemostasis, especially under stress conditions including acute colitis (Fig. 7). Gadd45β was strongly expressed in the small and large intestine and protected against DSS-induced colitis. Furthermore, Gadd45β is not regulated by the immune system but contributes to intestinal wound healing. Furthermore, Smad7 is a novel interacting partner of intestinal Gadd45β; this interaction inhibits the E3 ligase activity of Smurf, which degrades TGF-βR1. Recent clinical trials of an oral Smad7-specific antisense oligonucleotide (Mongersen, GED-0301) suggest a beneficial effect on IBD^43,44^. Therefore, our results indicate that targeting Gadd45β is a potentially effective therapeutic approach for treating IBD.

## Supplementary information

Supplementary information
