# Supplementary material for: Gadd45β promotes regeneration after injury through TGFβ-dependent restitution in experimental colitis
Source: Exp Mol Med. 2019 Oct 30;51(10):128. doi: 10.1038/s12276-019-0335-y (PMC6821912; doi:10.1038/s12276-019-0335-y)
Supplement: Supplementary file 1 — Supplementary information [file 12276_2019_335_MOESM1_ESM.pdf]

Supplementary information

Gadd45β promotes regeneration after injury through TGFβ-dependent restitution in experimental colitis

Jung Hwan Hwang[1, 2, #, *], Tae-Hwan Kim[3, #], Yong-Hoon Kim[1, 2], Jung-Ran Noh[1], Dong-Hee Choi[1], Kyoung-Shim Kim[1, 2], Eun-Young Lee[4], Byoung-Chan Kim[4], Myung Hee Kim[4], Ho Kim[5], Tae Geol Lee[6], Jong-Soo Lee[3, *], and Chul-Ho Lee[1, 2, *]

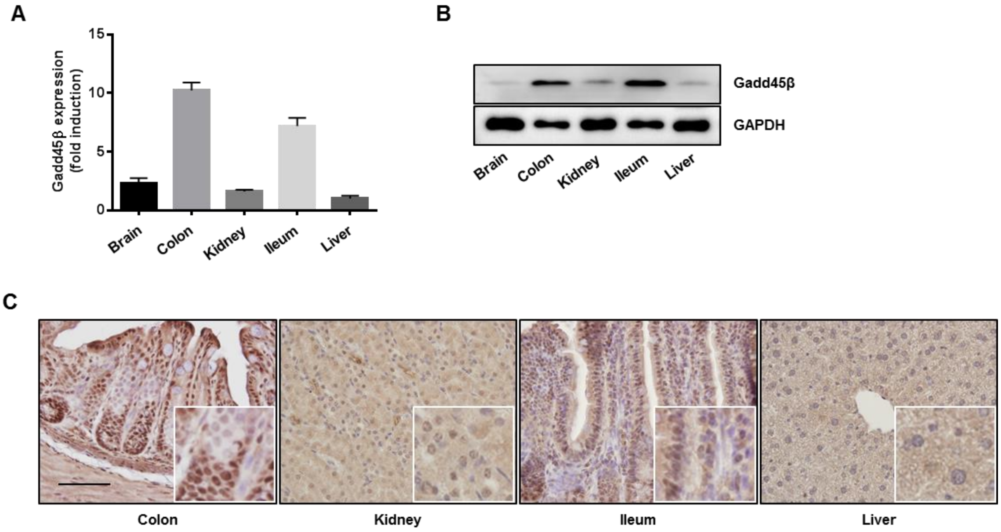

Supplementary Figure 1. mRNA expression, protein levels, and tissue distribution of Gadd45β. **a** The mRNA expression levels of *Gadd45β* in several tissues from C57BL/6J mice were estimated via quantitative real-time polymerase chain reaction (PCR). **b** Protein levels were evaluated via immunoblotting using a specific antibody against Gadd45β. **c** The tissue distribution of Gadd45β was determined via immunostaining using a specific antibody against Gadd45β. Bar represents 100 μm.

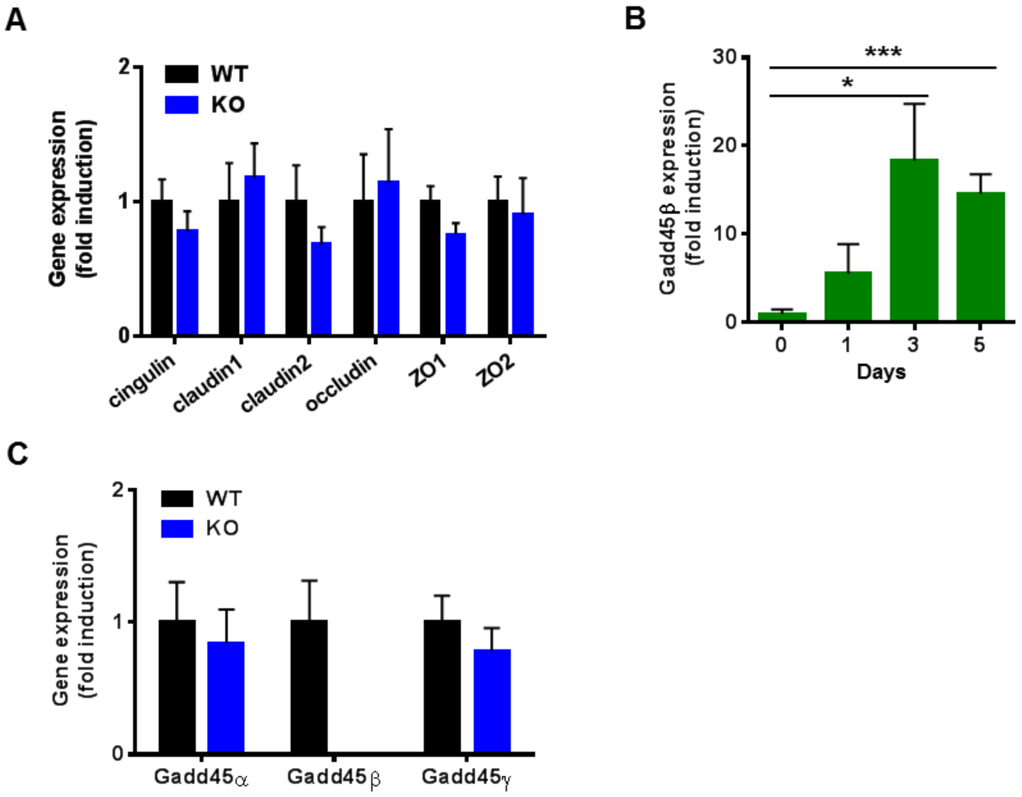

Supplementary Figure 2. a Expression levels of genes related to tight junction regulation were analyzed via real-time PCR in the colon of Gadd45β-WT (n = 5, black) and -KO mice (n = 5, blue) and levels are presented relative to those in WT mice. ZO1; zonula occludens1, ZO2; zonula occludens2. **b** Gadd45β expression in the colon at 0, 1, 2, 3, and 5 days after treatment with 3% DSS. C57BL/6 mice received 3% DSS in drinking water for the indicated time points. mRNA expression levels of *Gadd45β* in the colon were estimated by real-time PCR and mRNA levels at each time point are presented relative to level in the untreated group (0 days). Results are expressed as means ± SEM. $*P < 0.05$ or $***P < 0.001$. **c** Gene expression levels of *Gadd45α*, *Gadd45β*, and *Gadd45γ* were determined by real-time PCR in the colon from Gadd45β WT (n = 5) and KO (n = 5) mice. The results are presented relative to levels in WT mice.

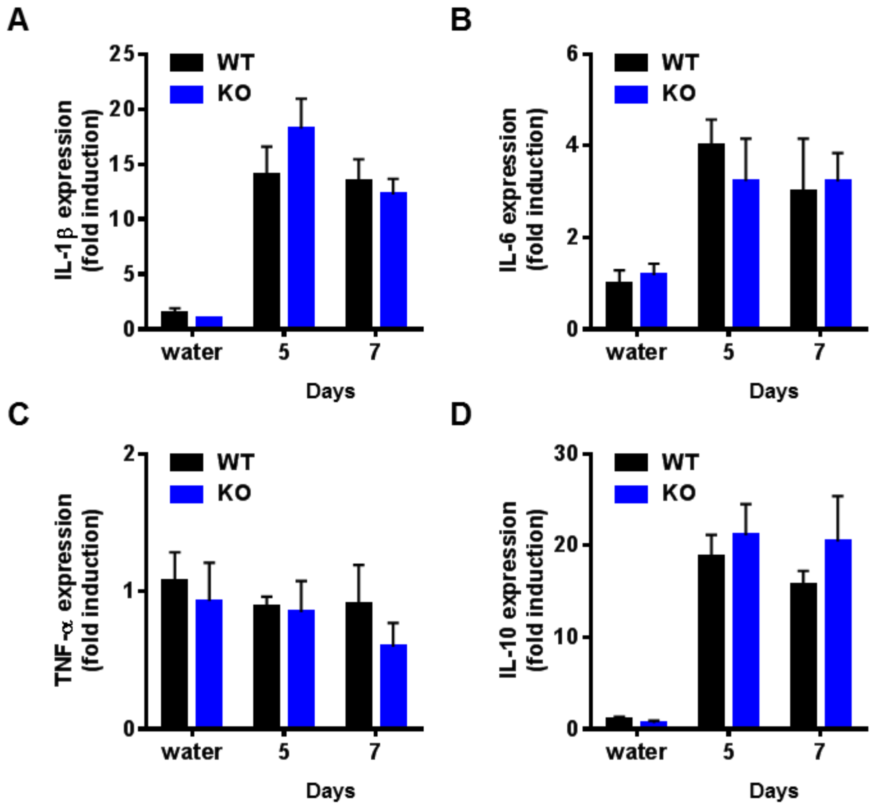

Supplementary Figure 3. a-d Expression levels of genes encoding cytokines, such as IL-1β, IL-6, TNF-α, and IL-10, were estimated in the colon of Gadd45β-WT (n = 3–5, black) and -KO (n = 3–5, blue) mice at 5 or 7 d after DSS treatment. mRNA levels are presented relative to those in WT mice.

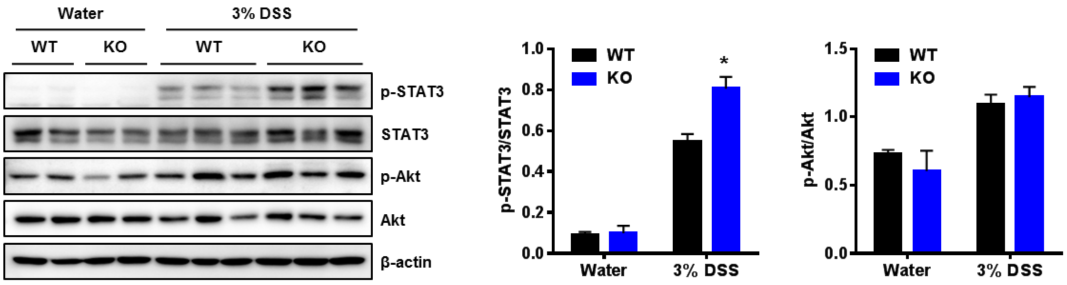

Supplementary Figure 4. Signaling pathways regulated by Gadd45β in the colon tissues. The active phosphorylation of Stat3 and Akt in the colon from Gadd45β WT and KO mice at 5 days after treatment with 3% DSS were determined by immunoblotting using specific antibodies against the indicated proteins and phosphorylated forms based on density. Results are expressed as means ± SEM. $*P < 0.05$.

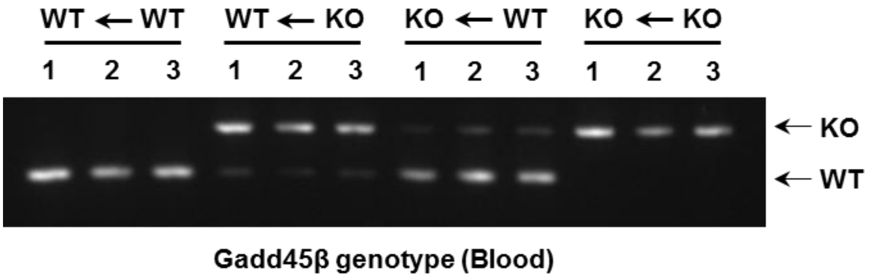

Supplementary Figure 5. Reconstitution of recipient mice with donor bone marrow. Representative bone marrow reconstitutions of recipient mice were estimated by PCR using specific primers and genomic DNA isolated in blood cells.

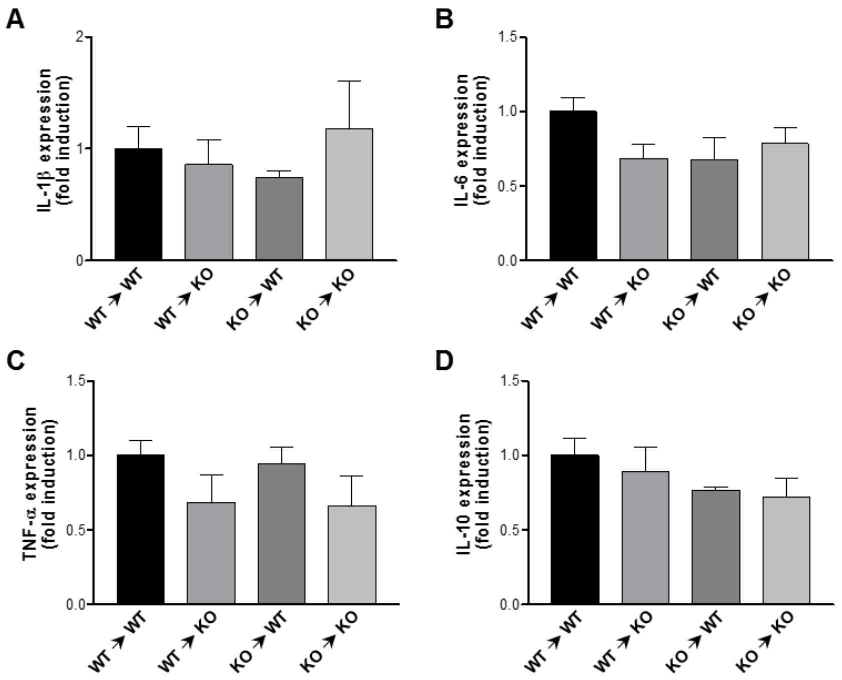

Supplementary Figure 6. a-d Expression levels of genes encoding cytokines, such as IL-1β, IL-6, TNF-α, and IL-10, were estimated in the colon of WT → WT (n = 5), WT → KO (n = 5), KO → WT (n = 5), and KO → KO (n = 5) groups at 5 days after treatment with 3% DSS. mRNA levels are presented relative to those in WT → WT group mice.

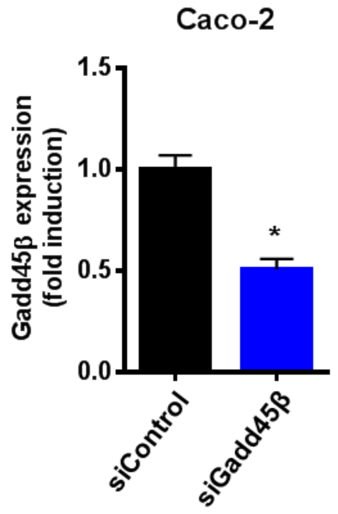

Supplementary Figure 7. Silencing efficacy of Gadd45β siRNA in intestinal cell lines. Caco2 cells were treated with control siRNA or specific Gadd45β siRNA 29 (20 nM) for 24 h and the efficacy was estimated with qRT-PCR. Results are expressed as means ± SEM. *$P < 0.05$.

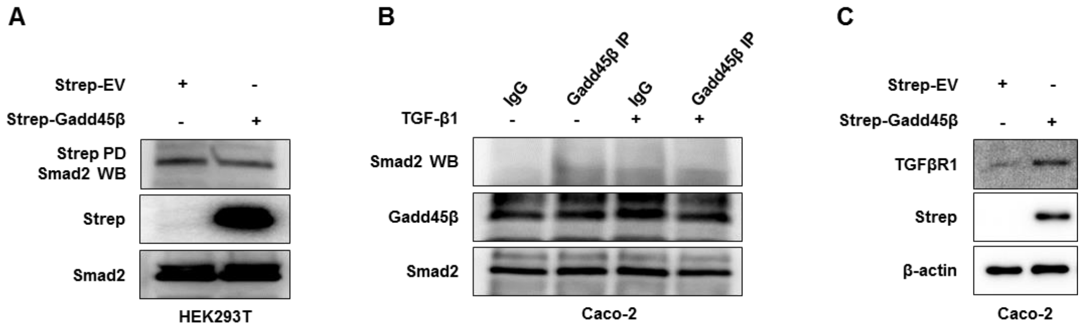

Supplementary Figure 8. Interaction of Gadd45β with Smad2 *in vitro*. **a** HEK293T cells were transfected with an empty vector or Strep-tagged Gadd45β, and cell lysates were used for a Strep pull-down assay, followed by immunoblotting with an anti-Smad2 antibody. Whole cell lysates were subjected to immunoblotting with the indicated antibodies. **b** Caco-2 cells were treated with mock or TGF-β1 (10 ng/mL) for 10 min, and cell lysates were subjected to immunoprecipitation with the IgG control or anti-Gadd45β antibody, followed by immunoblotting with an anti-Smad2 antibody. Whole cell lysates were subjected to immunoblotting with the indicated antibodies. **c** Caco-2 cells were transfected with a Strep-tagged Gadd45β plasmid for 1 d, and cell lysates were used for immunoblotting to determine the stability of TGF-βR1.

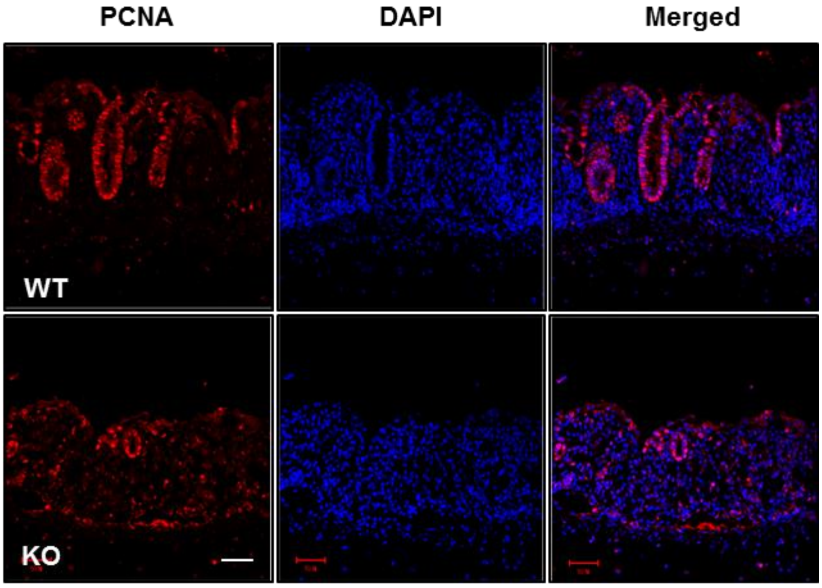

Supplementary Figure 9. Impaired proliferation of cells in the colon from Gadd45β KO mice. Gadd45β WT and KO mice received 3% DSS in drinking water for 5 days and were then permitted to recover by replacing DSS with regular drinking water. After 3 days of recovery, colon sections from WT and KO mice were stained with a primary antibody against PCNA (red) and then further incubated with a secondary antibody and DAPI (blue). Bar represents 50 nm.

Supplementary Table. List of primers of each gene used for quantification by qRT-PCR

Gene name	Primers
Cingulin	CTAAACCGACTTCCTCGATTAA
	TGTTGATGAGCGAGTCCACTG
Claudin1	GGCTTCTCTGGGATGGATCG
	TTTGCGAAACGCAGGACATC
Claudin2	CAGAGTGGCTGTAGTGGGTG
	GCGAGTAGAAGTCCCGAAGG
Occludin	CCAGGCAGCGTGTTCCT
	TTCTAAATAACAGTCACCTGAGGGC
ZO1	CCCTACCAACCTCGGCCTT
	AACGCTGGAAATAACCTCGTTC
ZO2	AGCACGCCCTGCTCGAC
	TCACGATTGGAAACCACTGAGT
Gadd45α	CTGCCAAGCTGCTCAACGTA
	ACGGATGAGGGTGAAATGGA
Gadd45β	CGGAGACATTGGGCACAAC
	CCTTGGCTTTTCCAGGAATCT
Gadd45γ	GCATTGCATCCTCATTTCGA
	CCTCGCAGAACAAACTGAGCTT
IL-1β	AGGAGCTATCACTTGACCACAT
	TGATGTGCTGCTGCGAGATT
IL-6	TCCATCCAGTTGCCTTCTTG
	TTCCACGATTTCCCAGAGAAC
TNFα	GGTCCCCAAAGGGATGAGAA
	TGAGGGTCTGGGCCATAGAA
IL-10	GGGTTGCCAAGCCTTATCG
	TCTCACCCAGGGAATTCAAATG
18s	GACACGGACAGGATTGACAGATTGATAG
	GTTAGCATGCCAGAGTCTCGTTCGTT
